# Guanine-based spin valve with spin rectification effect for an artificial memory element

**DOI:** 10.1016/j.heliyon.2024.e41171

**Published:** 2024-12-12

**Authors:** Nicusor Iacob, Cristina Chirila, Mama Sangaré, Andrei Kuncser, Anda E. Stanciu, Marcela Socol, Catalin C. Negrila, Mihaela Botea, Claudiu Locovei, Gabriel Schinteie, Aurelian C. Galca, Anca Stanculescu, Lucian Pintilie, Victor Kuncser, Bogdana Borca

**Affiliations:** a National Institute of Materials Physics, 077125 Magurele, Ilfov, Romania; b Institute of Applied Sciences, University of Sciences, Techniques and Technology of Bamako (USTTB), Bamako, Mali

**Keywords:** Guanine nucleobase, Multiferroic junction, Organic ferroelectric, Spin valve, Electroresistance, Magnetoresistance

## Abstract

Non-volatile electronic memory elements are very attractive for applications, not only for information storage but also in logic circuits, sensing devices and neuromorphic computing. Here, a ferroelectric film of guanine nucleobase is used in a resistive memory junction sandwiched between two different ferromagnetic films of Co and CoCr alloys. The magnetic films have an in-plane easy axis of magnetization and different coercive fields whereas the guanine film ensures a very long spin transport length, at 100 K. The non-volatile resistance states of the multiferroic spintronic junction with two-terminals are manipulated by a combined action of small external magnetic and electric fields. Thus, the magnetic field controls the relative orientation of the magnetization of the metallic ferromagnetic electrodes, that leads to different magnetoresistance states. The orientation and the magnitude of the electric field controls the orientation of the polarization of the guanine ferroelectric barrier, that leads to different electroresistance states, respectively. Moreover, we have observed a strong interfacial coupling of the two parameters. Consequently, positive and negative magnetoresistance hysteresis loops corresponding to spin rectification effects and non-hysteretic (erased) resistive states are manipulated with the electric field by switching the orientation of the electrical polarization of the organic ferroelectric.

## Highlights

• Artificial memory element consisting of a DNA nucleobase.
• Electrical polarization control of the multi-level magnetoresistance.
• Weak electric and magnetic fields for switching memresistive states.

## Graphical abstract

**Figure:**
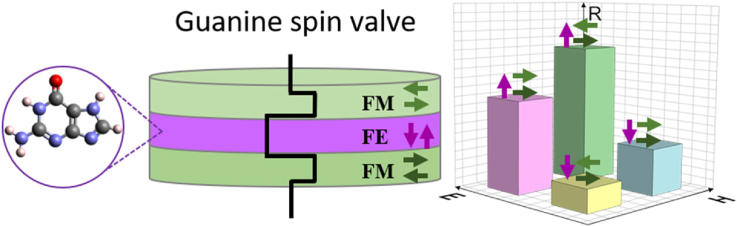

## 1 Introduction

The realization of electronic elements using organic molecules and biomolecules, including the nucleobases and the nucleic acids, has nowadays a major scientific and technological interest for applications in the fields of bioelectronics [1,2], molecular electronics and optoelectronics [[3], [4], [5], [6]], memory units and neuromorphic computing [7,8]. Therein, the multi-level resistive, *i.e.* memristive devices [9], which switch and retain their electrical resistance, can be used as computing units to store and process information [10], and can serve as models of the synaptic and neuronal operations [11,12]. Memristive behavior is reached in ferroelectric tunnel junctions [13,14] with multilevel values of the electroresistance (ER), which can be switched by the amplitude of the voltage pulse to configure the electrical polarization of the ferroelectric part [15]. Besides, memristive functions can also be realized in magnetic tunnel junctions where magnetoresistive states (MR) are depending on the relative orientation of the magnetization of the magnetic electrodes and can be switched using the magnetic field or the spin transfer torque effect of the spin-polarized current [16,17]. An additional degree of tuneability was proposed and implemented recently in systems by coupling the magnetic and electric functionalities in multiferroic tunnel junctions [[18], [19], [20], [21], [22]]. In this novel thematic, the use of organic materials is still a scarcely explored field [[23], [24], [25], [26], [27], [28], [29], [30]]. By our knowledge, the use in such devices of bio-molecules, that are biocompatible and/or have fundamental roles in the life of organisms, was never explored or demonstrated. In this context, new contributions are of great importance in material sciences and innovative technologies for green low-power consumption and biocompatible electronic applications.

The realization of the hybrid magneto-electric systems containing organic material apart from presenting various advantages related to their low cost, versatile preparation, flexibility, large area, lightweight and biocompatibility, have a combination of favorable properties for applications in spintronics and molecular electronics. Thus, the lightweight elements in their composition showing a small spin-orbit coupling, favor the preservation of the electron spin state for longer times than in standard inorganic materials, *i.e.* leading to longer spin lifetime and coherence of the charge carriers [31]. Based on the weak spin-orbit and weak hyperfine interactions [32], organic materials also present a long spin-transport length, in the range of hundreds of nanometers [[33], [34], [35], [36], [37]], compared with a few nanometers for inorganic materials. Therein, in a few examples are used organic tunnel barriers of Tris(8-hydroxyquinoline)-aluminum(III) (Alq_3_) [23], poly(vinylidene fluoride) [24,25] and poly(vinylindene fluoride-trifluoroethylene) (P(VDF-TrFE)) [26,27] intercalated between the magnetic electrodes such as La_2/3_Sr_1/3_MnO_3_ (LSMO) and Co [23,24,26,27] or Fe_2_O_3_ and Co [25]. These magnetic junctions show resistive switching of up to 4-5 orders of magnitude and magnetoresistance of 70 % arising through changes at the ferromagnetic/organic interfaces [23]. The tunneling electro-magneto-resistance can reach around 1000 % while the tunneling electroresistance reaches about 30 % at 200 K [27] by tuning gradually the interfacial spin polarization of the device and by controlling the ferroelectric polarization with an applied electric field. Similarly, spin-polarization of the PVDF/Co spinterface controlled by tuning the ferroelectric polarization of the PVDF was used as spin-filters for the electrons tunneling through PVDF to change thus the sign of the MR [24]. Besides, it was shown in these studies that factors as temperature and thickness of the ferroelectric tunnel barrier can be used to influence the values of the tunneling resistance and the spin diffusion length [25,26,30].

Here, we realize a multi-level resistive state multiferroic junction with two terminals that consists of a guanine nucleobase film sandwiched between ferromagnetic electrodes of Co and Co_85_Cr_15_ films that have in-plane easy axes of magnetization and different coercive fields. The molecular guanine films are ferroelectric, showing typical ferroelectric polarization–electric field hysteresis loops with a large electrical polarization at low temperatures of up to 200 K [38]. Above this transition temperature, at which different properties are affected, the guanine films have a preponderant paraelectric phase containing residual or locally induced nanoscopic ferroelectric domains, as observed by piezoresponse force microscopy at room temperature [38].

### 1.1 Experimental methods

The metallic electrodes and the commercially available synthetic guanine molecules (acquired from Acros Organics-purity: 99+%) were deposited by thermal evaporation in two different high vacuum chambers, respectively (with the base pressure in the range of 10^−6^ mbar). The substrate consists of in-house prepared Au films (occasionally used for contacts) with a Ti buffer layer (both films deposited by radio-frequency magnetron sputtering) on naturally oxidized float-zone silicon ⟨100⟩ ‘n’ type phosphorus-doped wafers. The bottom magnetic electrode, which generates the spin-polarized current, located under the organic film, consists of a Co_85_Cr_15_ film contacted a few mm laterally away below the top electrode contact. The top Co electrodes of the Co/guanine/Co_85_Cr_15_ system were prepared on a 1 mm^2^ grid masks. The Co films were covered by a thin protecting layer of Au. The stacking structure of the spin-valve magnetic junction was measured by Transmission Electron Microscopy (TEM) using a Cs-corrected JEOL ARM 200F instrument with Electron Energy Loss Spectroscopy (EELS) in the mass-thickness contrast mode.

The magneto-electrical measurements were realized in a two-terminal configuration at a temperature of 100 K using a Quantum Design Physical Property Measurement System PPMS in the AC transport mode, where an excitation current is applied trough the sample and a resulting potential difference is measured. The magnetoresistance data were measured with an excitation current amplitude of 0.01 mA, at 1 Hz frequency along a time window of 1.25 s and 1.75 s, for the different orientations of the polarization of the guanine film, respectively. For switching the polarization, electrical pulses corresponding to excitation currents of much higher amplitudes of up to 0.125 mA were applied with the above-mentioned time window, respectively. For all measurements the guanine film thickness was about 200 nm. The mean resistivity of the guanine film in the spin-valve junction obtained by magnetoresistive measurements is about 2.1x10^6^ Ω cm.

## 2 Results and discussions

**Fig. 1:**
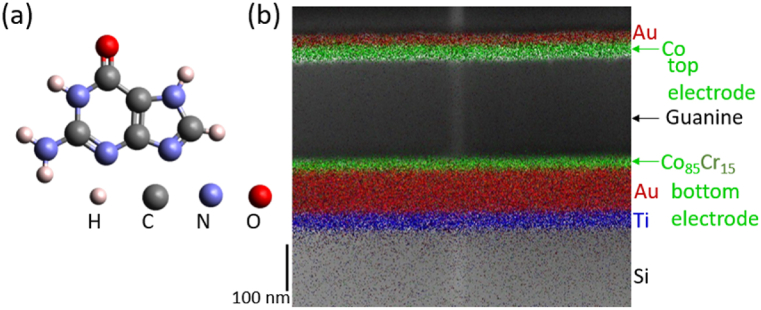
Two-terminal vertical multiferroic junctions with a ferroelectric guanine spacer. (a) Ball-and-stick model of the guanine molecule. (b) Scanning transmission electron microscopy of the multiferroic junction with the stacking structure of Au/Co/Guanine/Co_85_Cr_15_/Au/Ti/Si-substrate. The metal elementary maps colored with Au (red), Co (green), Ti (blue) are overlaid on top of the microscopic image.

The multi-level resistive system consists of a two terminals junction that is composed of a guanine nucleobase (that have the chemical formula C_5_N_5_H_5_O) film sandwiched between ferromagnetic electrodes of Co and Co_85_Cr_15_ films. The guanine film exhibits ferroelectric properties [38] and is used as a spin transport medium in the magnetic junction, enabling switching between distinct resistive states controlled by the orientation of its electric polarization. The molecular model and the TEM image of the stacking structure of the multi-layered junction with the metal elementary maps overlaid on top of the microscopic image are represented in Fig. 1(a) and (b), respectively.

**Fig. 2:**
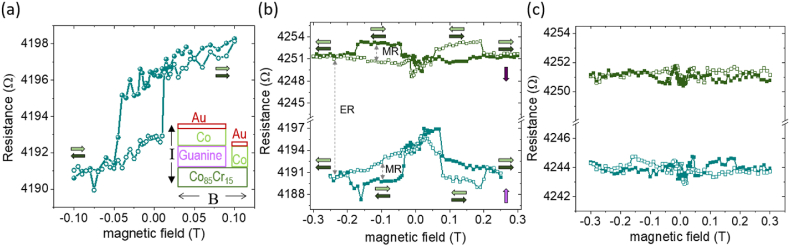
Electroresistive and magnetoresistive properties of the metal-organic multiferroic junction. (a) Minor loop of the magnetoresistance measured at 100 K in the as-cooled state of the junction. The arrows mark the relative orientation of the magnetization of the two magnetic films of Co (top arrow) and Co_85_Cr_15_ (bottom arrow). Inset: Schematic representation of the sample configuration, indicating the current flow and the applied magnetic field. (b) Major loops of magnetoresistance in the as-cooled state of a junction (bottom curve) and after switching the polarization with an electrical pulse (top curve). The polarization states of the barrier are marked with vertical arrows. The MR and ER values are marked with dashed arrows. (c) Erased magnetoresistance states of the loops from panel (b) obtained after applying different electric pulses for depolarization. Filled and open symbols are used for the two senses of the magnetic field ramps, from positive to negative and negative to positive, respectively.

The resistive results obtained in an as-cooled spontaneous polarization conformation of the guanine film, shows a spin-valve minor-loop magnetoresistive effect, under the application of a magnetic field (Fig. 2 (a)). The major-loops of magnetoresistance were obtained by increasing the range of the magnetic field (Fig. 2 (b)). The bottom curves correspond to the low resistive state obtained under the initial as-cooled electric polarization configuration and persists for applied electric pulses with excitation currents below 0.1 mA (1 Hz, 1.25 s). For a current amplitude of 0.125 mA of the electric pulses, the polarization state of the film is changed, which induces an increase of the mean resistance based on the electroresistive effect (Fig. 2 (b)-top curve). The ferroelectric control of the magnetoresistance relies on the electrostatic shift of energy levels by the change of the polarization that changes the electrical dipole at the interface [27]. The hysteresis loop shows a positive magnetoresistive effect (Fig. 2 (b)-top curves). In this case, the higher resistive values correspond to antiparallel magnetization alignment of the two ferromagnetic films and lower values correspond to parallel magnetization alignments. Fig. 2 (b)-bottom curves present a negative magnetoresistive effect, where the magnetoresistance has the lower resistance values corresponding to antiparallel magnetization alignment of the two electrodes and higher values for the parallel magnetization alignments. The monotone background reversed magnetoresistive effect, that may be encountered in organic spin valves [[39], [40], [41]] is most probably caused here by misaligned polarization domains in the as cooled ferroelectric state. As an additional support for the different electric polarization in the two cases are the higher resistance values in the top curves, as compared to the resistance values in the bottom curves. Thus, the poling of the sample induces the spin rectification effect. The large electrical polarization of the ferroelectric guanine film [38] enhances the electrostatic potential at the interface and its effect on the spin injection and spin transport properties, allowing thus, the change in sign of the magnetoresistive effect, *i.e.* the spin rectification [19,23,26,29,30]. Considering the rapport with the orientation of the polarization, the latest conformation corresponds to a high resistive state. The relatively low resistance values of the junction may be an effect of a rough interface between the top electrode and the organic spacer, which may shorten the actual thickness of the organic layer [42].

Furthermore, a full electrical control of the magnetoresistance implies the possibility of erasing the memristive states. Based on the fact that the depolarization field for the ferroelectric materials is undergoing the minor loops [43], we applied successive electrical pulses with decaying amplitude and alternating the period of 1.25 s and 1.75 s after each magnetoresistive hysteresis loops. The magnetoresistance measured after these procedures shows no hysteresis (Fig. 2 (c)). Therefore, the magnetoresistance is manipulated by applying electric fields. Different orientations and magnitudes of the electric field are producing the inversion [23,29] or, when the depolarization effects are dominant [44,45], even the elimination of the magnetoresistive hysteresis loops [46]. This ensures an essential issue for the information technologies, the erasure of the resistive states.

The magnetoresistance percentage ratio MR(%) is defined as: MR = (R_ap_-R_p_)/R_p_, where R_ap_ and R_p_ are the resistance values that correspond to an antiparallel and respectively a parallel orientation of the magnetization of the ferromagnetic films. Similarly, the electroresistance ER(%) is defined as ER=(R_down_-R_up_)/R_up_, where R_up_ and R_down_ represent the resistance levels for different polarizations orientations of the ferroelectric film. In Fig. 2 (b) are marked the MR and ER values. The high and low resistive states have similar negative and positive opening of the hysteretic MR of approximately 0.05 %, while the ER is approximately 1,43 %. The ON/OFF ratios of the magnetoresistance and electroresistance levels are all approximately 1.

**Fig. 3:**
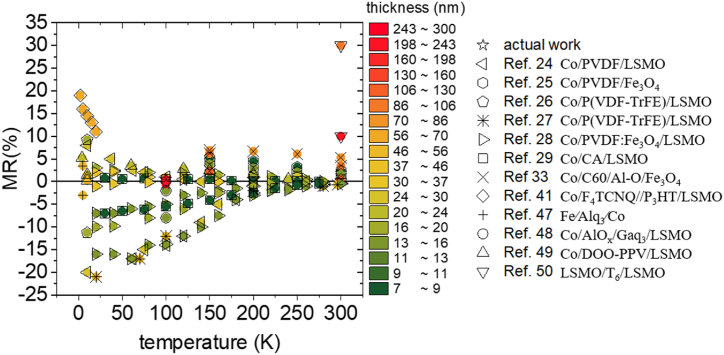
Magnetoresistance values of different organic multiferroic junctions and spin-valves in function of temperature and thickness. Bibliographic data are adapted from Refs. [[24], [25], [26], [27], [28], [29],^33^,^41^,[47], [48], [49], [50]]. The color scale represents the thickness of the organic films.

The MR value is affected by the system characteristics, such as the measurement temperature, the thickness of the molecular barrier, the degree of spin-polarized current generated through the ferromagnetic electrodes as well as the sharpness of the interfaces. MR strongly decreases by increasing the temperature and the thickness [30,35,36]. A comparison of MR with bibliographic results measured at various temperatures for organic spin valves containing organic ferroelectric [[24], [25], [26], [27], [28], [29]] and organic semiconductor [33,41,[47], [48], [49], [50]] films that have different thickness of the molecular layer, is presented in Fig. 3. Therefore, variations of these conditions may be considered for the enhancement of the MR. Additionally, a higher current's spin polarization will also ensure a higher MR effect. Thus, materials that have a very large spin polarization effect [[51], [52], [53]], may be elsewhere envisaged to be used for the ferromagnetic electrodes. Nevertheless, we have observed a long spin transport length at 100 K. This value includes our system among the molecular spintronic junctions with very long spin transport lengths [33,37,47]. Another factor that can decrease the MR is the shunting effect, typically manifested along the current-in-plane path when a conductive substrate or buffer layer is used at the interface with the magnetic electrode [54]. This current shunting effect can alter the MR of our junctions.

**Fig. 4:**
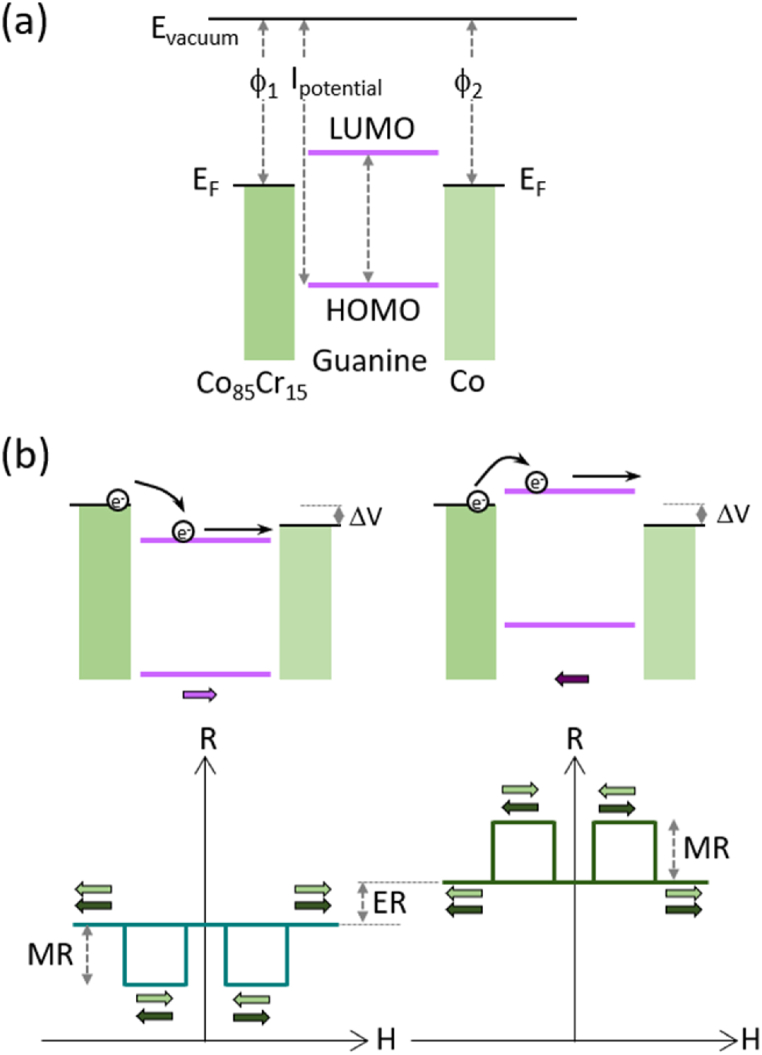
Energy levels alignment of the guanine spin valve and the mechanism of the electrically controlled spin rectification. (a) Simple model of the energy levels alignment of the Co/Guanine/Co_85_Cr_15_ heterojunction. (b) Electron transport mechanism for different electrical polarizations of the guanine film for an applied ΔV potential and the corresponding rectification effect of the magnetoresistive states (bottom panels).

The transport properties and thus, the magneto- and electro-resistive effects respectively, strongly depend on the metal-organic interfaces. Primarily, the energy levels alignment at the interfaces and in particular the relative alignment of the Fermi level (E_F_) with the molecular electronic states, defines the type of the transport carriers. By considering perfect metal-organic interfaces and the independent characteristics of each film, the Co/Guanine/Co_85_Cr_15_ heterojunction have the main carriers the electrons (Fig. 4 (a)), due to the nearness position of the E_F_ to the lowest unoccupied molecular orbital (LUMO). The work function (ϕ) of polycrystalline Co and Cr materials is 5 eV and 4.5 eV, respectively [55]. Guanine is a wide band gap semiconductor with the energy gap (E_g_) between the highest occupied molecular orbital (HOMO) and the LUMO determined experimentally of approximately 4 eV [38,56], whereas the ionization potential (I_potential_) is approximately 8 eV [57]. In addition, the electrostatic potential at the interfaces alters the electronic structure and defines the carrier injection barrier at the metal-organic interface. This is especially important for the ferroelectric spacers in spin valves [58], of which polarization involves the separation of positive and negative charges and generates an unequal electric potential in space and can actively control the interfacial properties, such as the electrochemical potential and the charge distribution. Thus, at the metal/ferroelectric interface, the screening charges that accumulate in the metal depend on the orientation of the polarization and on the metallic properties. Different metallic electrodes, with different work functions produce asymmetric electric potentials that cause the bending and shifts of the energy levels, which affect the spin and charge transport. Thus, the electrostatic shift of the energy levels allows the electrical control of the magnetoresistive states (Fig. 4 (b)).

Furthermore, the charge transport mechanism in organic systems involves the following scenarios: *(i)* tunneling from electrode to electrode, especially at low temperatures and for very thin films (mechanism that may be excluded for our systems), *(ii)* hopping from localized electronic states that could be the molecular orbitals in different layers of the film, which becomes dominant when is thermally activated *(iii)* band or transport channels that are delocalized electronic states formed by molecular interactions and wave-functions overlapping. In addition, the relative contributions of each mechanism depend, however, on microscopic parameters. Therefore, the molecular charge carriers' mobility in organic semiconductors is based not only on electronic but also on electron-phonon interactions, electronic and phonon bandwidth and high conductivities of the charge carriers are encountered especially in π-conjugated systems [59,60]. Moreover, an efficient overlapping of the wave-functions that depends on the molecular packing, can derive in transport channels that lead to very long charge carriers’ transport lengths. These effects were observed for DNA [61], but also predicted for guanine crystals [62]. Furthermore, our results show that guanine films are a favorable medium also for the spin propagation.

## 3 Conclusions

In summary, we demonstrate here the ferroelectrical control of the magnetoresistive states of a spin valve junction of Co/Guanine/Co_85_Cr_15_, that can be turned on and off between a number of non-volatile memristive states with small electric and magnetic fields. These states are written in positive and negative magnetoresistance hysteresis loops and erased with the electric fields by switching the orientation of the electrical polarization of the organic ferroelectric film. The guanine film ensures a very long spin transport length at 100 K. These results establish firstly that guanine nucleobase can be used in a synthetic memory element, and offer a promising platform for new systems of memristive junctions where the magnetic films may be replaced by magnetic 2D van der Waals materials [[63], [64], [65], [66]], or 2D-organic magnets [67] and organic spinterfaces [68] as electrodes. A few recent successful examples that already include this type of electrodes are photovoltaic [69], capacitive [70] and spin-crossover [71] devices.

## Funding

This work was funded by project PN-III-P2-2.1-PED-2021-0378 (contract no. 575PED/2022) of the Romanian Ministry of Research, Innovation and Digitalization through UEFSCDI as well as by the Core Program of the National Institute of Materials Physics under the Project PC2– PN23080202. V. Kuncser and G. Schinteie acknowledge financial support from EU under Romanian Recovery and Resilience Plan PNRR, Pillar III, Component C9-I8, contract nr.760083/23.05.2023. M. Sangaré acknowledges Eugen Ionescu fellowship program financed by the Romanian Ministry of Foreign Affairs and 10.13039/501100002708Agence Universitaire de la Francophonie (AUF).

## CRediT authorship contribution statement

**Nicusor Iacob:** Visualization, Validation, Investigation, Formal analysis. **Cristina Chirila:** Validation, Investigation, Formal analysis. **Mama Sangaré:** Visualization, Investigation, Formal analysis. **Andrei Kuncser:** Visualization, Investigation, Formal analysis. **Anda E. Stanciu:** Writing – original draft, Investigation, Formal analysis. **Marcela Socol:** Validation, Resources, Investigation. **Catalin C. Negrila:** Resources, Investigation. **Mihaela Botea:** Investigation. **Claudiu Locovei:** Investigation. **Gabriel Schinteie:** Investigation. **Aurelian C. Galca:** Resources, Investigation. **Anca Stanculescu:** Supervision, Project administration. **Lucian Pintilie:** Supervision, Project administration. **Victor Kuncser:** Supervision, Project administration, Conceptualization. **Bogdana Borca:** Writing – review & editing, Writing – original draft, Supervision, Project administration, Investigation, Funding acquisition, Formal analysis, Conceptualization.

## Data availability statement

Data will be made available on request.

## Declaration of competing interest

The authors have no conflicts to disclose.
